# Effect of sodium-glucose cotransporter 2 (SGLT2) inhibitors on left ventricular remodelling and longitudinal strain: a prospective observational study

**DOI:** 10.1186/s12872-021-02250-9

**Published:** 2021-09-21

**Authors:** Sergio Gamaza-Chulián, Enrique Díaz-Retamino, Fátima González-Testón, José Carlos Gaitero, María José Castillo, Raquel Alfaro, Elías Rodríguez, Eva González-Caballero, Antonio Martín-Santana

**Affiliations:** 1 Departmento de Cardiología, Hospital de Jerez de la Frontera, Cádiz. Departamento de Medicina, Universidad de Cádiz, Carretera Circunvalación s/n, Jerez de la Frontera, Cádiz, Spain; 2 Departmento de Cardiología, Hospital de Jerez de la Frontera, Cádiz, Spain; 3 Centro de Salud Barrio Bajo, Sanlúcar de Barrameda, Cádiz, Spain; 4 Centro de Salud La Milagrosa, Jerez de la Frontera, Cádiz, Spain; 5 Centro de Salud Jerez Centro, Jerez de la Frontera, Cádiz, Spain

**Keywords:** Diabetes mellitus, SGLT2 inhibitors, Cardiac remodelling, Speckle tracking echocardiography

## Abstract

**Background:**

Sodium-glucose cotransporter 2 inhibitors (SGLT2i) lower cardiovascular events in type 2 diabetes mellitus (T2DM) patients, although the mechanisms underlying these benefits are not clearly understood. Our aim was to study the effects of SGLT2i on left ventricular remodelling and longitudinal strain.

**Methods:**

Between November 2019 and April 2020, we included 52 patients with T2DM ≥ 18 years old, with HbA1c between 6.5 and 10.0%, and estimated glomerular filtration ≥ 45 ml/min/1.73 m^2^. Patients were classified into SGLT2i group and control group, according to prescribed treatment by their referring physician. Conventional and speckle tracking echocardiography were performed by blinded sonographers, at baseline and after 6 months of treatment.

**Results:**

Among the 52 included patients (44% females, mean age 66.8 ± 8.6 years, mean HbA1c was 7.40 ± 0.7%), 30 patients were prescribed SGLT2i and 22 patients were classified as control group. Mean change in indexed left ventricular mass (LVM) was − 0.85 ± 3.31 g/m^2^ (*p* = 0.003) in the SGLT2i group, and + 2.34 ± 4.13 g/m^2^ (*p* = 0.58) in the control group. Absolute value of Global Longitudinal Strain (GLS) increased by a mean of 1.29 ± 0.47 (*p* = 0.011) in the SGLT2i group, and 0.40 ± 0.62 (p = 0.34) in the control group. We did not find correlations between changes in LVM and GLS, and other variables like change in HbA1c.

**Conclusions:**

Among patients with T2DM, SGLT2i were associated with a significant reduction in indexed LVM and a significant increment in longitudinal strain measured by speckle tracking echocardiography, which may explain in part the clinical benefits found in clinical trials.

## Background

Sodium-glucose cotransporter 2 (SGLT2) inhibitors are a recent and fast growing class of oral anti-hyperglycaemic agents available to treat patients with type 2 diabetes (T2DM) [1]. They function through a novel mechanism by reducing renal tubular glucose reabsorption, and produce a reduction in blood glucose without stimulating insulin release. When compared with other oral anti-hyperglycaemic agents, SGLT2 inhibitors have demonstrated non-inferiority along with additional metabolic benefits of weight loss and blood pressure lowering [2]. In addition, SGLT2 inhibitors have been shown to improve cardiovascular outcomes in type 2 diabetes mellitus (T2DM) trials [3–6]. The mechanisms underlying the clinical cardiovascular beneficial effects, especially on heart failure, are not fully understood and have been the subject of various studies and publications [7, 8].

Reverse ventricular remodelling refers to a “more-normal” chamber geometry restoration [9]. Several pharmacological treatments such as angiotensin-converting enzyme (ACE) inhibitors [10], beta-blockers [11, 12] and mineralcorticoid receptor antagonists [13], have been shown to promote reverse ventricular remodelling, with reductions in left ventricular mass (LVM) and volume and improved left ventricular systolic function. These changes are consistently associated with reductions in morbidity and mortality. As a result, some authors advocate that reverse remodelling can serve as a valid surrogate endpoint for clinical outcomes in studies of new therapies [14].

Furthermore, Global Longitudinal Strain (GLS) determined by Speckle Tracking technique is a surrogate of left ventricular systolic function [15]. Clinical studies of the effects of SGLT2 inhibitors on myocardial deformation parameters are scarce [16, 17]. Although LV longitudinal strain was previously measured by cardiac magnetic resonance [18], to our knowledge, there are no studies estimating GLS by speckle tracking echocardiography in patients treated with SGLT2 inhibitors.

We hypothesised that the SGLT2 inhibitors effects on left ventricular remodelling may play a role in the underlying mechanisms through which SGLT2 inhibitors reduce the risk of heart failure in people with diabetes. Our aim was to study the effects of SGLT2 inhibitors on left ventricular remodelling and function in patients with T2DM.

## Methods

### Study design and participants

This was a prospective observational study conducted in a single centre in Jerez de la Frontera (Spain). Patients were recruited from the endocrinology outpatient department. Fifty-two consecutive diabetic patients were included prospectively. The inclusion criteria were: patients with T2DM with at least 18 years of age attending our clinic between November 2019 and April 2020; glycated haemoglobin levels between 6.5 and 10.0%; 3) Clinical stability. The exclusion criteria were: history of type 1 diabetes mellitus; current SGLT2 inhibitor or glucagon-like peptide receptor agonist use; an estimated glomerular filtration rate < 45 ml/min/1.73 m^2^; acute coronary syndrome the last 2 months; previous cardiac surgery; pregnant women; New York Heart Association IV symptoms of heart failure; greater than moderate valvular disease; or suboptimal echocardiographic acoustic window.

Clinical decisions on medical management were made by the referring physician based on clinical data and co-morbidities at baseline visit, according to current recommendations [19].

### Data collection and follow-up

Clinical, anthropometric, analytical and echocardiographic assessments were performed at baseline and after 6 month follow-up. Arterial blood pressure was also estimated during the initial visit. According to the prescribed treatment at this point, patients were classified into the SGLT2 inhibitors group or the control group. The same sonographers, who were blinded to clinical data, baseline echocardiographic data and prescribed treatment, performed both echocardiographic examinations.

### Variables

The primary outcome endpoint was the change in ventricular remodelling and function between initial and follow-up echocardiographic assessment.

### Standard echocardiographic examination

Two-dimensional trans-thoracic echocardiographic and Doppler studies were obtained with clinical ultrasound machines equipped with 2.5–3.5 MHz transducers (iE33 Phillips Medical Systems, The Best, The Netherlands). All tests were conducted by two experienced sonographers, who were blinded to the clinical data and prescribed treatment. Baseline echocardiographic examination was performed during the first 7 days after inclusion in the study.

Left ventricular chamber dimensions and wall thicknesses were measured, and left ventricular mass was calculated according to the American Society of Echocardiography guidelines [20]. Left ventricular hypertrophy (LVH) was defined as indexed left ventricular mass of 95 g/m^2^ or greater for women and 115 g/m^2^ or greater for men [20]. The relative wall thickness (RWT) was calculated as the ratio of posterior wall thickness/left ventricular diastolic radius, independently of the presence of LVH. A ratio of 0.42 or greater indicated concentric left ventricular geometry. End-diastolic and end-systolic left ventricular volumes were estimated and left ventricular ejection fraction (EF) was assessed by the modified Simpson’s Biplane Method. To assess diastolic function, the following mitral Doppler pulse and tissue Doppler variables were measured: early (E) and late (A) diastolic filling velocity, E/A ratio, septal (septal e’) and lateral (lateral e’) early mitral annular tissue velocity. We also calculated the E/e’ ratio.

**Fig. 1:**
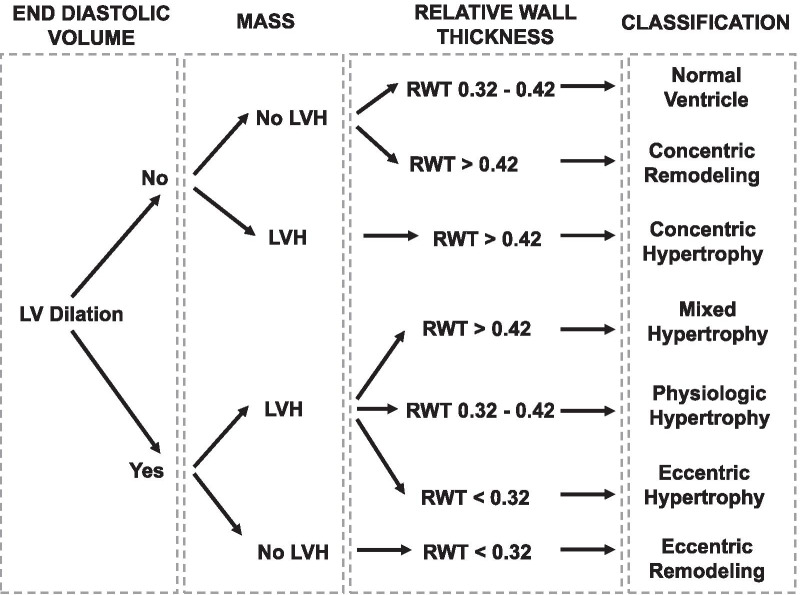
Left ventricular remodelling classification

According to LVVi (cut-off value 75 mL/m^2^), LVMi (cut-off value 115 g/m^2^ in men and 95 g/m^2^ in women), and RWT, patients were classified into 8 geometric patterns. Normal ventricle was considered as normal LVMi, normal LVVi, and RWT between 0.32 and 0.42. Dilated and hypertrophied ventricles were classified, according to RWT, as eccentric hypertrophy (RWT < 0.32), mixed hypertrophy (RWT > 0.42), or dilated hypertrophy (RWT 0.32–0.42). Non-dilated ventricles with RWT > 0.42 are categorized as having concentric remodelling or concentric hypertrophy, based on the value of LVMi. Dilated ventricles with normal LVMi and RWT < 0.32 are described as eccentric remodelling. Patients were classified into 8 geometric remodelling patterns according to the end-diastolic left ventricular volume (LVV) (cut-off value 75 ml/m^2^), LVH and RWT [21] (Fig. 1).

### Strain analyses

Myocardial strain was measured using Speckle Tracking echocardiography. To assess LV, longitudinal strain the endocardial and epicardial borders were traced in the apical two-, three- and four-chamber echocardiographic views on an end-diastolic frame. The software then automatically divided the myocardium into 17 segments. Peak systolic strain was estimated for each segment, and then GLS was calculated by averaging the 17 segments values. All images were stored electronically and LV strain was analyzed off-line with 2D Speckle Tracking software (QLab 10).

### Statistical analysis

Data were expressed as mean ± standard deviation for continuous variables, and were compared by using the unpaired *t*-test. Categorical variables were expressed as percentages and were compared using chi-square analysis or the Fisher exact test. Comparison of variables between baseline and 6 months after treatment was made using the paired test or Wilcoxon signed-rank test. Baseline and 6-month variables were also assessed by ANOVA for repeated-measures with time and group-time interaction effects. Comparisons between changes in indexed LVM, GLS and other continuous variables were calculated by Pearson correlation.

Analyses followed an intention-to-treat approach, where all patients were included in their corresponding group according to the initial prescribed treatment.

Differences were considered significant at *p* values < 0.05. For data analysis, the statistical program SPSS version 20.0 (SPSS Inc., Chicago, Illinois) was used.

## Results

### Baseline characteristics

A total of 52 patients (29 males and 23 females) were included in the study after exclusion of 2 patients because of suboptimal acoustic window. Mean age of the patients was 66.8 ± 8.6 years, mean duration of diabetes was 104 ± 101 months, mean glycated haemoglobin was 7.40 ± 0.7%. Of the participants, 65% had arterial hypertension, 13% were current smokers, 54% had dyslipidaemia, and 4 patients (8%) had coronary artery disease. At baseline, 29% were on DPP-4 inhibitors, 27% on insulin, 79% on metformin, 75% on RAAS inhibitors (angiotensin-converting enzyme inhibitors or angiotensin receptor blockers) and 9% on beta-blockers.

Of the total sample, 30 patients were prescribed SGLT2 inhibitors (67% empagliflozin, 17% dapagliflozin, 10% canagliflozin, 7% ertugliflozin), whilst the remaining 22 patients were included in the “control” group. Basal clinical characteristics of both groups are summarized in Table 1, and echocardiographic characteristics in Table 2. Patients prescribed SGLT2 inhibitors had significantly higher glycated haemoglobin and worse GLS; however, we did not find any other differences in basal characteristics between both groups.

**Table 1:** Basal clinical characteristics in SGLT2i and control group

Variable	SGLT2i (n = 30)	Control (n = 22)	*p* value
Female, n (%)	13 (43%)	10 (45%)	0.88
Age (years)	65.7 ± 8.7	68.2 ± 8.5	0.32
Arterial hypertension	18 (60%)	16 (73%)	0.34
Smokers	6 (20%)	1 (4%)	0.22
Dyslipidaemia	16 (53%)	12 (54%)	0.93
Coronary disease	3 (10%)	1 (4%)	0.33
Metformin	23 (77%)	18 (82%)	0.74
DPP4 inhibitors	8 (27%)	7 (32%)	0.68
Insulin	9 (30%)	5 (23%)	0.56
RAAS inhibitors	21 (70%)	17 (77%)	0.56
Beta-blockers	3 (10%)	2 (9%)	0.89
Aldosterone antagonist	2 (7%)	0 (0%)	0.50
GF ≥ 60 ml/min/1.73 m^2^	12 (43%)	11 (50%)	0.24
Glycated haemoglobin	7.78 ± 0.94	6.97 ± 0.44	0.002
Diabetes duration (months)	87 ± 97	128 ± 105	0.14
BMI (kg/m^2^)	30.5 ± 6.8	28.9 ± 4.7	0.33
GF (ml/min/1.73 m^2^)	92.6 ± 40.8	86.7 ± 28.9	

*DPP4* dipeptidyl peptidase-4, *RAAS* renin-angiotensin-aldosterone system, *GF* glomerular filtration, *BMI* Body Mass Index

**Table 2:** Basal echocardiographic characteristics in SGLT2i and control group

Variable	SGLT2i (n = 30)	Control (n = 22)	*p* value
LV end-diastolic diameter (mm)	46.9 ± 5.0	44.5 ± 4.3	0.08
LV end-diastolic volume (ml)	103.3 ± 40.0	97.0 ± 25.7	0.52
LV indexed end-diastolic volume (ml/m^2^)	52.6 ± 18.9	50.2 ± 9.7	0.59
LV end-systolic volume (ml)	40.7 ± 26.5	33.9 ± 8.5	0.19
LV ejection fraction (%)	62.9 ± 8.2	64.6 ± 5.8	0.43
LV ejection fraction < 50%	2 (7%)	0 (0%)	0.50
E wave (cm/s)	72.2 ± 25.5	64.2 ± 19.7	0.22
A wave (cm/s)	82.8 ± 18.9	87.5 ± 19.2	0.41
LA indexed volume (ml/m^2^)	31.9 ± 9.8	28.7 ± 9.1	0.23
Lateral e’ (cm/s)	8.3 ± 2.2	8.3 ± 3.2	0.95
Septal e’ (cm/s)	5.9 ± 1.4	5.8 ± 1.2	0.73
E/A ratio	0.83 ± 0.39	0.81 ± 0.26	0.80
E/e’ ratio	10.5 ± 3.6	9.8 ± 2.4	0.40
LV indexed mass (g/m^2^)	98.5 ± 27.9	90.8 ± 21.0	0.28
RWT	0.47 ± 0.07	0.49 ± 0.08	0.29
GLS	− 17.8 ± 2.9	− 19.6 ± 2.5	0.02

*LV* left ventricular, *LA* left atrial, *RWT* relative wall thickness, *GLS* global longitudinal strain

Left ventricular remodelling was similar in both groups during the initial examination: concentric remodelling was the most frequent finding in the SGLT2 and control group (40.0% vs. 45.5%), concentric hypertrophy (27% vs. 27%) and normal geometry (23% vs. 27%).

### Outcome

At 6-month visit, 3 patients in the SGLT2 group had stopped this treatment during the follow-up (one patient one month after the first visit and two patients 5 months after the baseline examination) because of minor side effects. Three patients in control group initiated treatment with SGLT2 inhibitors, as indicated by their referring physician during follow-up (one after 1 month, one after 3 months and one other 5 months after the initial assessment).

**Fig. 2:**
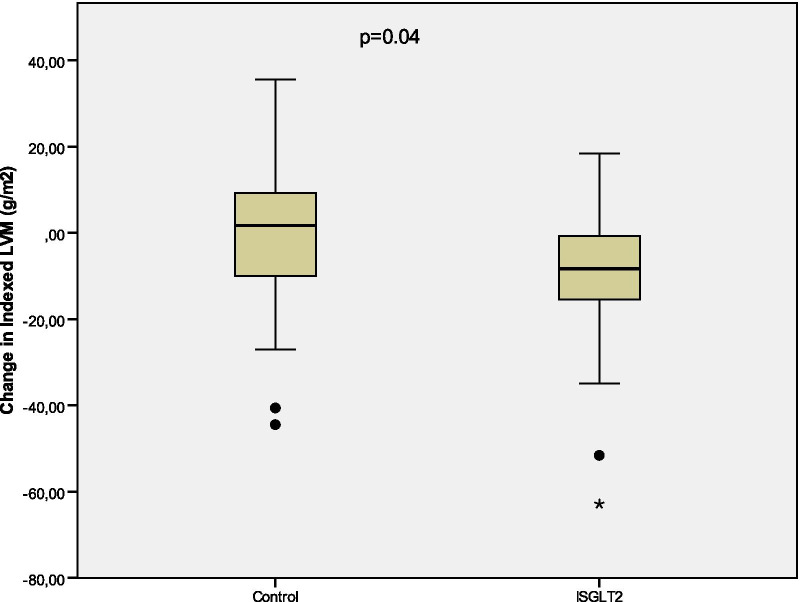
Change in indexed LVM from baseline to 6-month visit

**Fig. 3:**
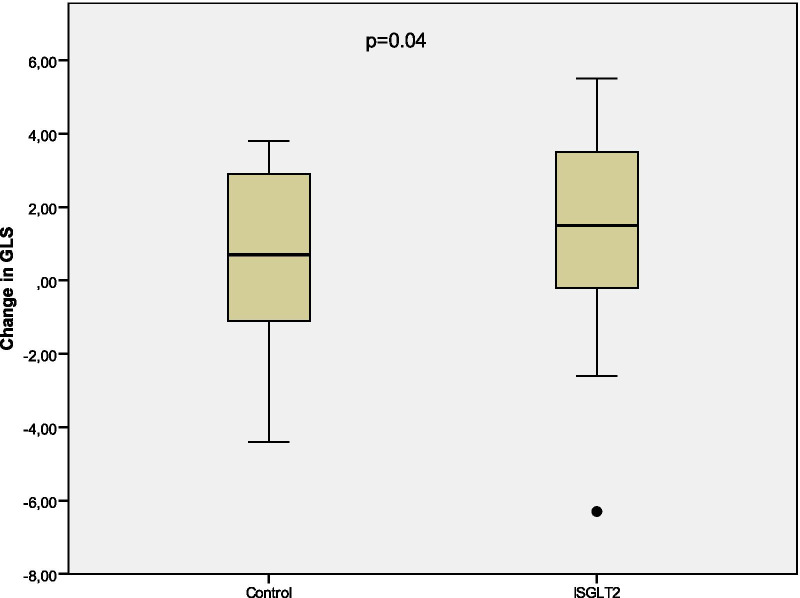
Change in GLS from baseline to 6-month visit

Mean change in the indexed LVM from baseline to the 6-month visit was − 10.85 ± 3.31 g/m^2^ (*p* = 0.003) in the SGLT2 group, and + 2.34 ± 4.13 g/m^2^ (*p* = 0.58) in the control group (Fig. 2). Absolute value of GLS increased by a mean of 1.29 ± 0.47 (*p* = 0.011) from baseline to the 6-month examination in the SGLT2 group, and 0.40 ± 0.62 (*p* = 0.34) in the control group (Fig. 3). Tables 3, 4 and 5 summarize the changes from baseline to 6-month visit.

**Table 3:** Changes from baseline to 6-month visit in SGLT2 patients

	Baseline	6-month	Δ from baseline	*p*
Indexed LVM (g/m^2^)	98.5 ± 27.9	87.6 ± 18.4	− 10.8 ± 3.3	0.003
LV end-diastolic volume (ml)	103.3 ± 40.0	100.8 ± 34.0	− 2.5 ± 5.0	0.62
LV end-diastolic diameter (mm)	46.9 ± 5.0	45.7 ± 4.3	− 1.2 ± 0.67	0.09
LV ejection fraction (%)	62.9 ± 8.2	62.6 ± 8.4	− 0.4 ± 1.5	0.81
RWT	0.47 ± 0.07	0.46 ± 0.06	− 0.01 ± 0.01	0.44
GLS	− 17.8 ± 2.9	− 19.1 ± 3.1	1.29 ± 0.47	0.01
LA indexed volume (ml/m^2^)	31.9 ± 9.8	31.2 ± 9.9	− 0.75 ± 1.28	0.56
E/A ratio	0.81 ± 0.39	0.92 ± 0.52	0.10 ± 0.05	0.07
Lateral e’ (cm/s)	8.31 ± 2.19	9.07 ± 2.57	0.76 ± 0.47	0.12
Septal e’ (cm/s)	5.91 ± 1.37	6.57 ± 1.60	0.66 ± 0.25	0.01
E/e’ ratio	10.5 ± 3.6	10.0 ± 3.9	− 0.52 ± 0.53	0.34

*LVM* left ventricular mass, *LV* left ventricular, *RWT* relative wall thickness, *GLS* global longitudinal strain, *LA* left atrial

**Table 4:** Changes from baseline to 6-month visit in control group

	Baseline	6-month	Δ from baseline	*p*
Indexed LVM (g/m^2^)	90.8 ± 21.0	88.5 ± 22.5	− 2.3 ± 4.1	0.58
LV end-diastolic volume (ml)	97.0 ± 25.7	96.2 ± 26.2	− 0.8 ± 4.7	0.86
LV end-diastolic diameter (mm)	44.5 ± 4.3	45.0 ± 4.2	0.4 ± 0.8	0.57
LV ejection fraction (%)	64.6 ± 5.8	64.4 ± 4.6	− 0.2 ± 1.3	0.89
RWT	0.49 ± 0.08	0.47 ± 0.10	− 0.02 ± 0.02	0.35
GLS	− 19.6 ± 2.5	− 20.0 ± 2.4	− 0.4 ± 0.6	0.34
LA indexed volume (ml/m^2^)	28.7 ± 9.1	28.9 ± 8.5	0.3 ± 1.4	0.85
E/A ratio	0.81 ± 0.26	0.73 ± 0.20	− 0.08 ± 0.04	0.05
Lateral e’ (cm/s)	8.26 ± 3.24	8.35 ± 2.35	0.09 ± 0.73	0.90
Septal e’ (cm/s)	5.78 ± 1.22	5.95 ± 1.26	0.16 ± 0.23	0.48
E/e’ ratio	9.79 ± 2.44	9.10 ± 2.36	− 0.69 ± 0.68	0.32

*LVM* left ventricular mass, *LV* left ventricular, *RWT* relative wall thickness, *GLS* global longitudinal strain, *LA* left atrial

**Table 5:** Comparisons between baseline and 6-month variables by repeated-measures ANOVA

Variable	Group (*p* value)	Time (*p* value)	Group-time (*p* value)
Indexed LVM (g/m^2^)	0.56	0.01	0.04
LV end-diastolic volume (ml)	0.53	0.64	0.81
LV end-diastolic diameter (mm)	0.19	0.48	0.12
LV ejection fraction (%)	0.32	0.79	0.93
RWT	0.35	0.21	0.70
GLS	0.02	0.005	0.04
LA indexed volume (ml/m^2^)	0.27	0.80	0.60
E/A ratio	0.36	0.74	0.01
Lateral e’ (cm/s)	0.52	0.31	0.42
Septal e’ (cm/s)	0.30	0.03	0.18
E/e’ ratio	0.31	0.16	0.83
Glycated haemoglobin	0.29	< 0.001	0.22

*LVM* left ventricular mass, *LV* left ventricular, *RWT* relative wall thickness, *GLS* global longitudinal strain, *LA* left atrial

**Fig. 4:**
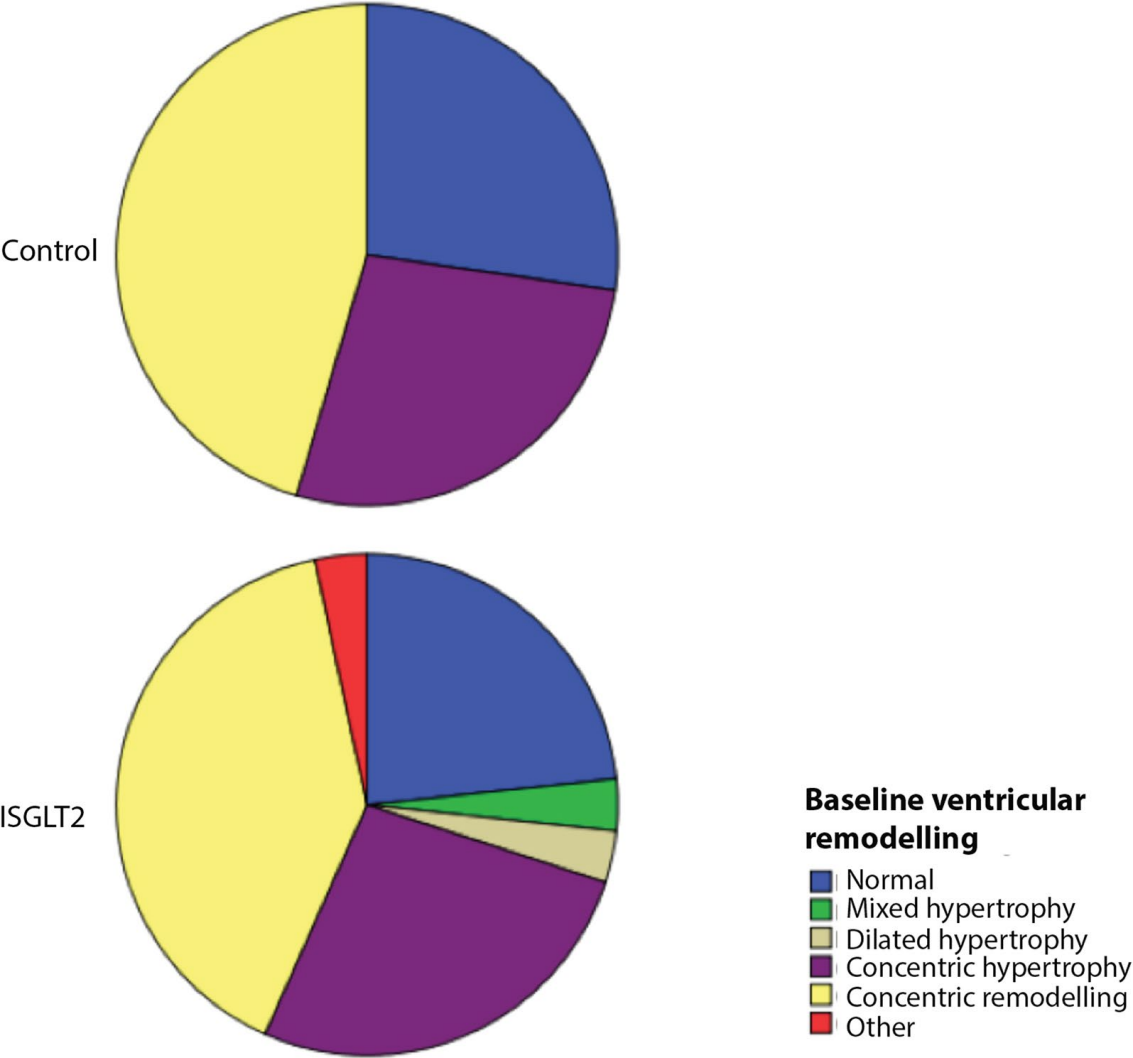
Baseline ventricular remodelling in control and SGLT2 groups

**Fig. 5:**
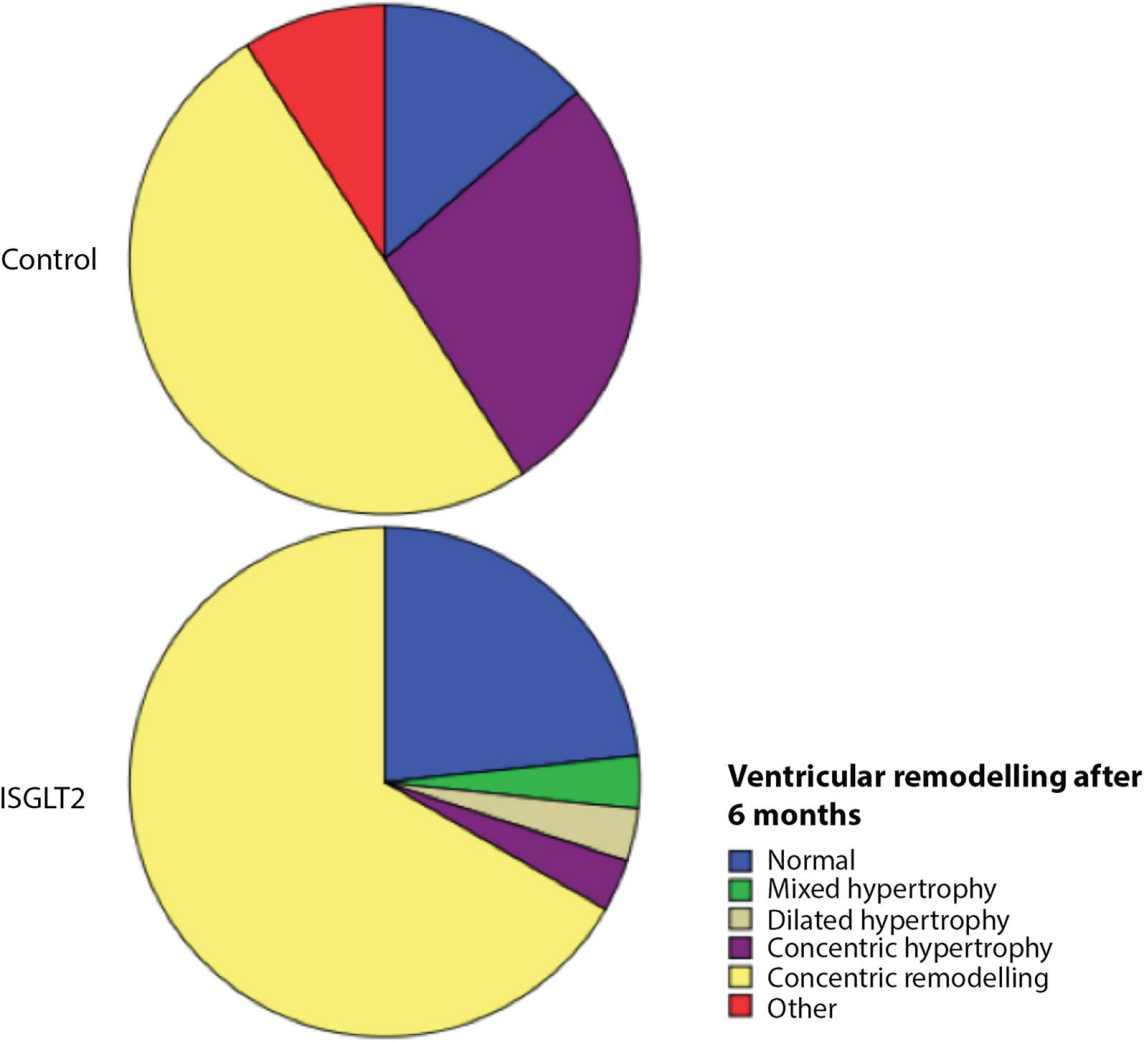
Ventricular remodelling after 6 months of follow-up in control and SGLT2 groups

Ventricular remodelling classification did not change significantly in the control group after 6 months of follow-up. Nevertheless, 7 patients in the SGLT2 group changed from concentric hypertrophy to concentric remodelling due to a significant reduction in the indexed left ventricular mass (Figs. 4, 5), indicating that the concentric hypertrophy decreased during the follow-up (from 33 to 10%, *p* = 0.006).

Glycated haemoglobin decreased in both groups: in SGLT2 patients from 7.8 ± 0.9% at baseline, to 5.8 ± 2.7% during the 6-month visit (-1.9 ± 2.8%, *p* = 0.001) and in the control group from 6.9 ± 1.0% to 5.9 ± 2.5% (− 1.0 ± 2.5%, *p* = 0.07).

Glomerular filtration did not change significantly in the control group (from 85.2 ± 29.0 to 82.6 ± 28.9, *p* = 0.47), whilst there was a non-significant increase in patients treated with SGLT2 inhibitors (from 87.6 ± 32.0 to 90.6 ± 29.9, *p* =  0.19).

We failed to find any correlations between changes in LVM, GLS, septal e’ and other variables (Table 6).

**Table 6:** Univariate correlates of change in indexed LVM, GLS and septal e’ vs. baseline and changes in variables

Variable	Δ indexed LVM		Δ GLS		Δ septal e’	
	r	*p*	r	*p*	r	*p*
Age (years)	− 0.06	0.69	0.22	0.11	− 0.11	0.46
Basal glycated haemoglobin	− 0.16	0.25	− 0.19	0.17	0.13	0.36
Δ glycated haemoglobin	− 0.06	0.65	− 0.10	0.46	0.23	0.08
Duration of diabetes (months)	0.01	0.92	− 0.01	0.93	− 0.18	0.21
GF (ml/min/1.73 m^2^)	0.07	0.62	− 0.26	0.06	0.23	0.12
Δ GF (ml/min/1.73 m^2^)	− 0.09	0.55	− 0.04	0.78	0.15	0.36
SBP (mmHg)	− 0.18	0.21	0.21	0.13	0.20	0.16
DBP (mmHg)	− 0.12	0.39	− 0.09	0.53	− 0.07	0.63
LV ejection fraction (%)	− 0.04	0.78	0.02	0.90	− 0.03	0.82
Δ Weight (Kg)	0.07	0.63	− 0.09	0.51	0.22	0.13

*LVM* left ventricular mass, *GLS* global longitudinal strain, *Δ* change from baseline to 6-month visit, *GF* glomerular filtration, *SBP* systolic blood pressure, *DBP* diastolic blood pressure, *LV* left ventricular

## Discussion

The main findings of this study were that the addition of SGLT2 inhibitors to standard anti-hyperglycaemic treatment in people with T2DM was associated with: (1) a significant decrease in indexed LVM; (2) an improvement in left ventricular GLS assessed by speckle tracking echocardiography.

Although SGLT2 inhibitors have demonstrated a reduction in heart failure outcomes [3–6], even in non-diabetic patients [22] mechanisms to explain the cardiovascular benefits of these drugs are not clearly understood. Our data support the theory that the benefits of SGLT2 inhibitors are, at least in part, mediated via a mechanism independent of its glucose-lowering activity.

LV hypertrophy is a strong determinant of cardiovascular outcomes and mortality in the general population [23] and also in people with T2DM [24]. Several studies showed previously significant reductions in LVM in mice with [25] and without T2DM [26, 27]. In clinical research, two small-sized-sample studies found that empagliflozin [28] and canagliflozin [29] reduced LVM, although these studies were not controlled by placebo. Verma et al. [30] showed that mean LVM regression assessed by cardiac magnetic resonance after 6 months of treatment with empagliflozin in patients with coronary artery disease was 2.6 g/m^2^. Similarly, treatment with dapagliflozin reduced LVM measured by cardiac resonance [31]. In our study, we found a higher reduction in LV hypertrophy, probably due to different inclusion criteria, higher baseline LVM and overestimation of LV hypertrophy by echocardiography [32].

LVM reduction supposed a change in ventricular remodelling classification in SGLT2 inhibitors patients that could have an impact on cardiovascular outcomes [33].

One of the strengths of our study was that, to our knowledge, this is the first clinical study to show an improvement in absolute value of GLS estimated by speckle tracking echocardiography in patients treated with SGLT2 inhibitors. Speckle tracking echocardiography is a relatively new method used to measure systolic myocardial function, with higher prognostic value than LV ejection fraction [15].

Several studies observed that diabetic patients have lower absolute GLS values despite normal LV ejection fraction [34, 35]. Other authors suggested that GLS by speckle tracking echocardiography might detect changes in systolic function earlier than conventional methods [36], which could explain why other studies did not find differences in LV ejection fraction in T2DM patients [28–30].

Garcia-Ropero et al. [37] found that empagliflozin improved myocardial deformation estimated by speckle tracking echocardiography in an ischemic non-diabetic porcine model. However, clinical studies of the effects of SGLT2 inhibitors on myocardial deformation parameters are lacking. Tanaka et al. [38] showed a GLS enhancement in patients treated with dapagliflozin, with similar results to our study, although this study was not a placebo-controlled one.

The mechanisms of the beneficial effects of SGLT2 inhibitors on cardiac remodelling and function are not completely understood. Improved glycaemic control and hypotensive effects seem unlikely, given that their benefits would have taken years. Other hypotheses like intravascular volume reduction, inhibition in the Na/H exchanger, and tissue oxygenation improvement via increased haematocrit have been proposed [39]. It has also been postulated that SGLT2 inhibitors may increase myocardial energy supply and metabolic efficiency, thereby, improving myocardial performance. Santos-Gallego et al. [40] showed that empagliflozin switched myocardial fuel utilization away from glucose towards other molecules like ketone bodies, free fatty acids and branched-chain amino acid that improved myocardial energetics.

Although other authors demonstrated that SGLT2 inhibitors improve diastolic function in T2DM patients [29, 41, 42] we achieved a significant improvement only on septal e’ values (Table 4), probably due to our reduced sample size also without signification when using ANOVA analysis (Table 5).

In our opinion, our main limitation was the non-randomized design of our study that hampered the establishment of a cause-effect relationship between SGLT2 inhibitors and positive effects on LV mass and function. However, there is a biologic plausibility for a relation between our results and the positive clinical impact of SGLT2 inhibition on patients with heart failure and reduced ejection fraction both with and without T2DM [43]. Other limitations of our study were the short duration of the follow-up and the reduced number of patients. These limitations made it difficult to obtain statistically significant differences in other variables. In addition, unfortunately, the small sample size hampered the comparisons between different iSGLT2. However, despite the limited number of patients and relatively short follow-up, it seems that there are large differences in significant variables between the groups taking into account SGLT inhibitors as a whole. Finally, although cardiac magnetic resonance is the gold standard for cardiac chambers volume and mass assessment, we preferred the use of echocardiography due to a more widespread use. On the other hand, one strength of the study was that it evaluated the effects of SGLT2 inhibitors on LV mass and function in real-world settings. It included a patient population that may be more representative of the non-selective population normally used in randomised controlled trials and provided evidence that the treatment may exert positive effects in the every day practice.

## Conclusions

The present study showed that T2DM patients treated with SGLT2 inhibitors displayed positive effects on left ventricular remodelling due to a reduction in LVM, and LV longitudinal function assessed by speckle tracking echocardiography. These findings could explain the beneficial effects on cardiovascular outcomes seen in clinical trials.

## Data Availability

The datasets used/and analysed during the current study are available from the corresponding author on reasonable request.

## Abbreviations

GLS: global longitudinal strain
LVM: left ventricular mass
SGLT2: sodium-glucose cotransporter 2
T2DM: type-2 diabetes mellitus

## References

[1] Padhi S, Nayak AK, Behera A (2020). Type II diabetes mellitus: a review on recent drug based therapeutics. Biomed Pharmacother.

[2] Hsia DS, Grove O, Cefalu WT (2017). An update on sodium-glucose co-transporter-2 inhibitors for the treatment of diabetes mellitus. Curr Opin Endocrinol Diabetes Obes.

[3] Zinman B, Wanner C, Lachin JM, Fitchett D, Bluhmki E, Hantel S (2015). Empagliflozin, cardiovascular outcomes, and mortality in type 2 diabetes. N Engl J Med.

[4] Neal B, Perkovic V, Mahaffey KW, de Zeeuw D, Fulcher G, Erondu N (2017). Canagliflozin and cardiovascular and renal events in type 2 diabetes. N Engl J Med.

[5] Wiviott SD, Raz I, Bonaca MP, Mosenzon O, Kato ET, Cahn A (2019). Dapagliflozin and cardiovascular outcomes in type 2 diabetes. N Engl J Med.

[6] Cannon CP, Pratley R, Dagogo-Jack S, Mancuso J, Huyck S, Masiukiewicz U (2020). Cardiovascular outcomes with ertugliflozin in type 2 diabetes. N Engl J Med.

[7] Chin KL, Ofori-Asenso R, Hopper I, von Lueder TG, Reid CM, Zoungas S (2019). Potential mechanisms underlying the cardiovascular benefits of sodium glucose cotransporter 2 inhibitors: a systematic review of data from preclinical studies. Cardiovasc Res.

[8] Verma S, McMurray JJV (2018). SGLT2 inhibitors and mechanisms of cardiovascular benefit: a state-of-the-art review. Diabetologia.

[9] Kim GH, Uriel N, Burkhoff D (2018). Reverse remodelling and myocardial recovery in heart failure. Nat Rev Cardiol.

[10] Pfeffer MA (1994). Mechanistic lessons from the SAVE study. Survival and ventricular enlargement. Am J Hypertens.

[11] Groenning BA, Nilsson JC, Sondergaard L, Fritz-Hansen T, Larsson HB, Hildebrandt PR (2000). Antiremodeling effects on the left ventricle during beta-blockade with metoprolol in the treatment of chronic heart failure. J Am Coll Cardiol.

[12] Hall SA, Cigarroa CG, Marcoux L, Risser RC, Grayburn PA, Eichhorn EJ (1995). Time course of improvement in left ventricular function, mass and geometry in patients with congestive heart failure treated with beta-adrenergic blockade. J Am Coll Cardiol.

[13] Zannad F, Gattis Stough W, Rossignol P, Bauersachs J, McMurray JJV, Swedberg K (2012). Mineralocorticoid receptor antagonists for heart failure with reduced ejection fraction: integrating evidence into clinical practice. Eur Heart J.

[14] Saraon T, Katz SD (2015). Reverse remodeling in systolic heart failure. Cardiol Rev.

[15] Stanton T, Leano R, Marwick TH (2009). Prediction of all-cause mortality from global longitudinal speckle strain: comparison with ejection fraction and wall motion scoring. Circ Cardiovasc Imaging.

[16] Rau M, Thiele K, Hartmann N-UK, Schuh A, Altiok E, Möllmann J (2021). Empagliflozin does not change cardiac index nor systemic vascular resistance but rapidly improves left ventricular filling pressure in patients with type 2 diabetes: a randomized controlled study. Cardiovasc Diabetol.

[17] Yu Y-W, Zhao X-M, Wang Y-H, Zhou Q, Huang Y, Zhai M (2021). Effect of sodium–glucose cotransporter 2 inhibitors on cardiac structure and function in type 2 diabetes mellitus patients with or without chronic heart failure: a meta-analysis. Cardiovasc Diabetol.

[18] Lee MMY, Brooksbank KJM, Wetherall K, Mangion K, Roditi G, Campbell RT (2021). Effect of empagliflozin on left ventricular volumes in patients with type 2 diabetes, or prediabetes, and heart failure with reduced ejection fraction (SUGAR-DM-HF). Circulation.

[19] Cosentino F, Grant PJ, Aboyans V, Bailey CJ, Ceriello A, Delgado V (2020). 2019 ESC guidelines on diabetes, pre-diabetes, and cardiovascular diseases developed in collaboration with the EASD. Eur Heart J.

[20] Lang RM, Badano LP, Mor-Avi V, Afilalo J, Armstrong A, Ernande L (2015). Recommendations for cardiac chamber quantification by echocardiography in adults: an update from the American Society of Echocardiography and the European Association of Cardiovascular Imaging. J Am Soc Echocardiogr.

[21] Gaasch WH, Zile MR (2011). Left ventricular structural remodeling in health and disease: with special emphasis on volume, mass, and geometry. J Am Coll Cardiol.

[22] Santos-Gallego CG, Vargas-Delgado AP, Requena-Ibanez JA, Garcia-Ropero A, Mancini D, Pinney S (2021). Randomized trial of empagliflozin in nondiabetic patients with heart failure and reduced ejection fraction. J Am Coll Cardiol.

[23] Krumholz HM, Larson M, Levy D (1995). Prognosis of left ventricular geometric patterns in the Framingham Heart Study. J Am Coll Cardiol.

[24] Dawson A, Morris AD, Struthers AD (2005). The epidemiology of left ventricular hypertrophy in type 2 diabetes mellitus. Diabetologia.

[25] Joubert M, Jagu B, Montaigne D, Marechal X, Tesse A, Ayer A (2017). The sodium-glucose cotransporter 2 inhibitor dapagliflozin prevents cardiomyopathy in a diabetic lipodystrophic mouse model. Diabetes.

[26] Desjardins J, Zhang Y, Thai K, Kabir G, Gilbert R, Connelly K (2017). Empagliflozin reduces LV mass and improves diastolic function in an experimental model of heart failure with preserved EF. Can J Cardiol.

[27] Connelly KA, Zhang Y, Visram A, Advani A, Batchu SN, Desjardins J-F (2019). Empagliflozin improves diastolic function in a nondiabetic rodent model of heart failure with preserved ejection fraction. JACC Basic Transl Sci.

[28] Verma S, Garg A, Yan AT, Gupta AK, Al-Omran M, Sabongui A (2016). Effect of empagliflozin on left ventricular mass and diastolic function in individuals with diabetes: an important clue to the EMPA-REG OUTCOME trial?. Diabetes Care.

[29] Matsutani D, Sakamoto M, Kayama Y, Takeda N, Horiuchi R, Utsunomiya K (2018). Effect of canagliflozin on left ventricular diastolic function in patients with type 2 diabetes. Cardiovasc Diabetol.

[30] Verma S, Mazer CD, Yan AT, Mason T, Garg V, Teoh H (2019). Effect of empagliflozin on left ventricular mass in patients with type 2 diabetes mellitus and coronary artery disease: the EMPA-HEART cardiolink-6 randomized clinical trial. Circulation.

[31] Brown AJM, Gandy S, McCrimmon R, Houston JG, Struthers AD, Lang CC (2020). A randomized controlled trial of dapagliflozin on left ventricular hypertrophy in people with type two diabetes: the DAPA-LVH trial. Eur Heart J.

[32] Seo H-Y, Lee S-P, Park J-B, Lee JM, Park E-A, Chang S-A (2015). Discrepancies in Left ventricular mass calculation based on echocardiography and cardiovascular magnetic resonance measurements in patients with left ventricular hypertrophy. J Am Soc Echocardiogr.

[33] Pugliese NR, Fabiani I, La Carrubba S, Conte L, Antonini-Canterin F, Colonna P (2017). Classification and prognostic evaluation of left ventricular remodeling in patients with asymptomatic heart failure. Am J Cardiol.

[34] Nakai H, Takeuchi M, Nishikage T, Lang RM, Otsuji Y (2009). Subclinical left ventricular dysfunction in asymptomatic diabetic patients assessed by two-dimensional speckle tracking echocardiography: correlation with diabetic duration. Eur J Echocardiogr.

[35] Ng ACT, Delgado V, Bertini M, van der Meer RW, Rijzewijk LJ, Shanks M (2009). Findings from left ventricular strain and strain rate imaging in asymptomatic patients with type 2 diabetes mellitus. Am J Cardiol.

[36] Zoroufian A, Razmi T, Taghavi-Shavazi M, Lotfi-Tokaldany M, Jalali A (2014). Evaluation of subclinical left ventricular dysfunction in diabetic patients: longitudinal strain velocities and left ventricular dyssynchrony by two-dimensional speckle tracking echocardiography study. Echocardiography.

[37] Garcia-Ropero A, Santos-Gallego CG, Vargas-Delgado AP, Requena-Ibanez JA, Picatoste B, Ishikawa K (2020). Correlation between myocardial strain and adverse remodeling in a non-diabetic model of heart failure following empagliflozin therapy. Expert Rev Cardiovasc Ther.

[38] Tanaka H, Soga F, Tatsumi K, Mochizuki Y, Sano H, Toki H (2020). Positive effect of dapagliflozin on left ventricular longitudinal function for type 2 diabetic mellitus patients with chronic heart failure. Cardiovasc Diabetol.

[39] Lan NSR, Fegan PG, Yeap BB, Dwivedi G (2019). The effects of sodium-glucose cotransporter 2 inhibitors on left ventricular function: current evidence and future directions. ESC Heart Fail.

[40] Santos-Gallego CG, Requena-Ibanez JA, San Antonio R, Ishikawa K, Watanabe S, Picatoste B (2019). Empagliflozin ameliorates adverse left ventricular remodeling in nondiabetic heart failure by enhancing myocardial energetics. J Am Coll Cardiol.

[41] Soga F, Tanaka H, Tatsumi K, Mochizuki Y, Sano H, Toki H (2018). Impact of dapagliflozin on left ventricular diastolic function of patients with type 2 diabetic mellitus with chronic heart failure. Cardiovasc Diabetol.

[42] Shim CY, Seo J, Cho I, Lee CJ, Cho I-J, Lhagvasuren P (2021). Randomized, controlled trial to evaluate the effect of dapagliflozin on left ventricular diastolic function in patients with type 2 diabetes mellitus: the IDDIA Trial. Circulation.

[43] Rosano G, Quek D, Martínez F (2020). Sodium-glucose co-transporter 2 inhibitors in heart failure: recent data and implications for practice. Card Fail Rev.

